# The Narrative of a Recovery from Tic Douloureux

**Published:** 1838-10

**Authors:** 


Art. III.—*The Narrative of a Recovery from Tic Douloureux.* By the Rev. C. E. Hutchinson.—*London,* 1838. 8vo. pp. 43.

This is an interesting and even affecting narrative, drawn up from the purest motives of benevolence and conveyed in a style of elegance and simplicity, and without a particle of affectation or pretension. The author, a clergyman of the established church, is himself the subject of the case, which seems to have been a very severe one but with nothing very uncommon in its character. The disease lasted four years, with considerable intervals of ease; the site of the pain being chiefly the arm and hand. After trying almost every remedy that has been recommended in neuralgia, with at most very temporary benefit, the patient finally proceeded to Carlsbad and drank the waters for about six weeks, with partial relief. His attacks, however, continued, although in a mitigated form, until the following year (1832) when he took a course of the factitious Carlsbad at Brighton; since which time he has had no return of his disease. We have no doubt that both the true and the factitious waters were beneficial in this case; but it is by no means a strong instance of their efficacy. The author overlooks the possible influence of other agencies, especially change of climate and lapse of time; and he mentions incidentally a circumstance that occurred in the spring immediately succeeding the relief obtained in the autumn at Brighton, which circumstance might in the opinion of some explain the permanency of that relief; and had it occurred at an earlier period of the treatment, might, possibly, have stamped in the author's mind the remedy immediately preceding it, with all the preeminence which he now gives to Carlsbad. "In the spring following (he says) I lost a considerable quantity of blood from the hæmorrhoidal vessels. From this time I can date a very sensible improvement in my general health." (p. 29.) Although this pamphlet is written for non-professional readers, it will interest the medical practitioner also; and we recommend it to both classes, with confidence. It is very unlike most of the productions of lay instructors in the medical art.

